# Unraveling the Role of Leptin in Liver Function and Its Relationship with Liver Diseases

**DOI:** 10.3390/ijms21249368

**Published:** 2020-12-09

**Authors:** Maite Martínez-Uña, Yaiza López-Mancheño, Carlos Diéguez, Manuel A. Fernández-Rojo, Marta G. Novelle

**Affiliations:** 1 Hepatic Regenerative Medicine Group, Madrid Institute for Advanced Studies in Food (IMDEA-Food), CEI-UAM+CSIC, 28049 Madrid, Spain; maite.martinez@imdea.org (M.M.-U.); yaiza.lopez@imdea.org (Y.L.-M.); manuel.fernandez@imdea.org (M.A.F.-R.); 2 Center for Research in Molecular Medicine and Chronic Diseases, CIMUS, University of Santiago de Compostela-Instituto de Investigación Sanitaria (IDIS), 15782 Santiago de Compostela, Spain; carlos.dieguez@usc.es; 3 CIBER Fisiopatología de la Obesidad y Nutrición (CIBERobn), Instituto de Salud Carlos III, 28029 Madrid, Spain; 4 School of Medicine, The University of Queensland, Herston, 4006 Brisbane, Australia

**Keywords:** leptin, adipose tissue, non-alcoholic fatty liver disease (NAFLD), non-alcoholic steatohepatitis (NASH), hepatocarcinoma (HCC), partial hepatectomy, liver regeneration

## Abstract

Since its discovery twenty-five years ago, the fat-derived hormone leptin has provided a revolutionary framework for studying the physiological role of adipose tissue as an endocrine organ. Leptin exerts pleiotropic effects on many metabolic pathways and is tightly connected with the liver, the major player in systemic metabolism. As a consequence, understanding the metabolic and hormonal interplay between the liver and adipose tissue could provide us with new therapeutic targets for some chronic liver diseases, an increasing problem worldwide. In this review, we assess relevant literature regarding the main metabolic effects of leptin on the liver, by direct regulation or through the central nervous system (CNS). We draw special attention to the contribution of leptin to the non-alcoholic fatty liver disease (NAFLD) pathogenesis and its progression to more advanced stages of the disease as non-alcoholic steatohepatitis (NASH). Likewise, we describe the contribution of leptin to the liver regeneration process after partial hepatectomy, the mainstay of treatment for certain hepatic malignant tumors.

## 1. Introduction

The effects of leptin were first observed by studying inheritable obese phenotype in the ob/ob and db/db mouse lines [1,2]. Classical parabiosis studies conducted in these animal models by Hervey [3,4] and mainly by Coleman [5,6] provided the first evidence of the existence of a circulating factor able to indicate the amount of adipose energy store in the brain in order to maintain it relatively constant. However, it was not until 1994, when the product of the ob gene was sequenced, identified and named as leptin, from the Greek root *leptos* for “thin”. Later in 1995, the leptin receptor was described [7], and then subsequently, it was shown that this leptin receptor is encoded by the db gene and has multiple forms, one of which is defective in Coleman’s originally described db/db mice [8,9].

The identification of Coleman’s “satiety factor” and its receptors caused a great revolution in the physiological knowledge about the regulation of energy homeostasis. This fact opened a broad window to understand the pathophysiology of obesity and its comorbid conditions, such as non-alcoholic fatty liver disease (NAFLD) or an increased risk of developing liver cancer [10,11,12,13]. Today, it is well known that leptin is normally released in proportion to body fat [14], with a significant drop under fasting conditions [15,16], and that it exerts its action on the central nervous systems (CNS), both on a specific set of hypothalamic neurons, NPY/AgRP and POMC, to inhibit food intake and to increase energy expenditure, leading to a reduction in body weight [17,18,19], and on other extrahypothalamic areas [20,21]. Besides this anorexigenic effect, leptin plays an important role in a wide variety of physiological functions, including lipid and glucose metabolism, fertility and immune function, among others. All the leptin physiological actions and its molecular targets are beyond this manuscript, but excellent reviews on this topic have been written over these years and can be found elsewhere [22,23,24,25,26,27,28].

The alarming pandemic of metabolic syndrome has resulted in NAFLD becoming one of the most common causes of chronic liver disease worldwide, with a prevalence of over 25% and a major cause of liver-related morbidity and mortality [29,30]. Although NAFLD begins with liver lipid accumulation, this pathology comprises a continuum of liver abnormalities varying in severity. In fact, there is now enough evidence that NAFLD is a multisystemic disease, affecting extra-hepatic organs and regulatory pathways [31,32,33,34,35,36]. Interestingly, while leptin displays anti-steatotic properties as shown by the benefices of leptin treatment in lipodystrophy [37], which will be commented on through this manuscript, high plasma levels are associated with inflammatory and fibrogenic processes and possibly oncogenic effects on the liver. All these facts make leptin a key therapeutic target.

Despite all the studies conducted and the multiple attempts to optimize its application into the clinic during the latter quarter-century, the complete characterization of leptin’s biological functions still remains incomplete. Likewise, many of the metabolic, cancer and liver-related complications of the metabolic syndrome remain to be assessed and are necessary to better understand the mechanistic basis of leptin in this disease. Throughout this narrative review, we briefly summarize the main aspects concerning the essential communication of leptin with the CNS and its role in the metabolic and physiological processes within the liver. Following, we cover current knowledge about the implication of leptin in the development of the most prevalent metabolic liver diseases, from NALFD to hepatocellular carcinoma (HCC). Moreover, we focus on understanding the possible effects of leptin in liver regeneration after partial hepatectomy. Finally, we discuss future directions that should be taken to address some of the remaining questions.

## 2. Leptin Signaling in Liver Metabolism: The Physiological Role

In retrospective, and taking into account that the existence of leptin was postulated long before its discovery, the relevant physiological role of leptin in liver function was known even before leptin was discovered, since some of the cardinal features of both the ob/ob and the db/db mice exhibited marked alterations in liver function including impaired lipid tolerance, dyslipidemias and steatosis [38]. Some of these alterations were reversed to some extent following leptin administration to ob/ob mice. Although clearly, these data indicate the need for leptin or leptin-receptor (LEPR) mediated signaling to ensure adequate liver function, it was unclear whether leptin effects could be exerted through direct or indirect mechanisms. However, the fact that restricted feeding in ob/ob mice was less effective than exogenous leptin administration in reversing some of these abnormalities supported the potential existence of leptin’s direct liver effects [39,40,41]. Indeed, this possibility was supported by the fact that leptin receptors were present in hepatocytes and mediating some in vitro leptin effects. Hence, specific deletion of the liver’s leptin receptor led to increased insulin sensitivity, increased liver´s triglycerides (TG) content and lipid accumulation [42]. Despite these clear-cut biological effects, leptin exerts a plethora of effects in different organs, which may influence metabolic homeostasis. Among the different systems implicated, one that is currently the focus of attention is the CNS. The vast majority of the metabolic functions of leptin are mediated by LEPRs in the CNS. Within the CNS, the hypothalamus is a key region for the control of liver function, including leptin action via sympathetic and parasympathetic mechanisms, even though new brain areas, as well as non-neural cells, are also involved. The existence of efferent and afferent pathways between liver and CNS provided the basis for the CNS-liver axis, an axis that may also be influenced by the adipose tissue, both WAT (white adipose tissue) and BAT (brown adipose tissue) via adipokines and batokines, respectively, or signals generated at gastrointestinal level [20,43,44,45]. Most of these issues are out of the scope of this review in which we just focus on some specific aspects related to leptin effects on general glucose and lipid homeostasis because of their impact on liver function and related pathologies.

### 2.1. Leptin Effects Mediated by the CNS

**Figure 1:**
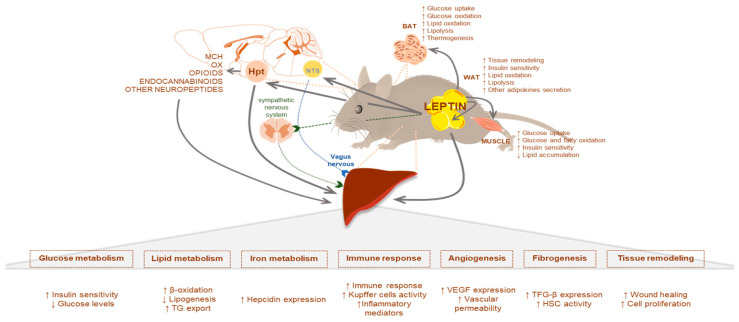
Adipocyte-derived leptin modulates hepatic metabolism through direct or indirect mechanisms. Shown is a schematic diagram of major leptin-regulate pathways by increasing (↑) or decreasing (↓) their activity.

Leptin modulation of the CNS activity is fascinating and complex. In addition to its direct action on neuronal pathways, leptin is able to regulate peripheral metabolism by acting through other neuropeptides [46,47,48] or via the autonomic nervous system. Hence, besides its direct action, some of the indirect leptin effects are explained below. Leptin acts synergistically with the vagal actions of the anorexigenic gut peptides like GLP-1 and cholecystokinin (CCK) [46]. In this context, the incretin GLP1 has also been proposed as a NAFLD treatment target since it increases hepatic fatty acid oxidation and decreases lipogenesis [49], while CCK plays an important role in the uptake of TG-derived fatty acids [50] and in the regulation of bile acid, which accumulation (cholestasis) reduces liver steatosis [51]. Several types of neurons in the lateral hypothalamus area (LHA) also respond to leptin and exert functions on liver metabolism. Indeed, leptin inhibits orexin (OX) neurons by multiple GABA (γ-aminobutyric acid)-independent mechanisms [52]. Recently, it has been described that a long-term OX-A overactivity due to lack of leptin signaling contributes to hepatosteatosis through an orexin/endocannabinoid/melanocortin interaction [53]. Despite these pieces of evidence, the interaction between leptin and orexins is complex, and further investigation will be needed to have a completed overview [54]. In this same context, leptin also inhibits melanin-concentrating hormone (MCH), a neuropeptide selectively expressed in the LHA. Interestingly, Imbernon et al. demonstrated that central MCH in the LHA modulated hepatic liver metabolism via the parasympathetic nervous system. After MCH infusion in mice, there was an increased TG content in the liver and in the activity of hepatic LPL (lipoprotein lipase), while other enzymes that control the hepatic lipoprotein assembly and secretion remained unchanged [55]. Nutrient partitioning is a sophisticated mechanism under the control of hormonal and neural signals [56]. Leptin exerts an essential role in this process either by acting directly or by modulating other neuropeptide actions, as it has previously been commented. Therefore, any dysregulation in one of these pathways can trigger a pathophysiological condition (Figure 1).

### 2.2. Leptin and Glucose Homeostasis

Leptin exerts its effects on glucose homeostasis by acting in a wide variety of tissues. These include intestinal mucosa (decreasing glucose absorption in the preprandrial state and the opposite postprandially), pancreas (decreasing insulin and glucagon secretion), suprarenal gland (decreasing glucocorticoids secretion), pituitary (increasing growth hormone secretion), muscle and BAT (increasing glucose uptake and glucose oxidation), WAT (decreasing insulin signaling and glucose metabolism) or liver (hepatic glucose flux). All these glucoregulatory effects are mostly mediated by hypothalamic pathways in the CNS, and they were revised quite recently by others being beyond the scope of this review [27,57,58,59]. The majority of results show that leptin in rodents enhances insulin-mediated suppression of hepatic glucose production; indeed, leptin and insulin have overlapping effects and may involve similar mechanisms [60]. However, the effects of leptin on liver glucose flux pathways are conflicting. It has been observed that leptin promotes and decreases hepatic glycogen storage and also promotes and suppresses hepatic gluconeogenesis, highlighting the complexity of leptin´s control of hepatic glucose metabolism. It is likely that some of this complexity derives from the effects dependent on factors such as nutritional status, fed vs. fasting, diet, adiposity, physical exercise, aging or circadian rhythms, to name a few [27,61]. This complexity is boosted by the fact that leptin biological effects are exerted at different tissues, including peripheral and central tissues. The latter is of relevance, taking into account that leptin bioavailability at the central level is a process highly regulated through the blood–brain barrier. Leptin entry into the CNS is via a saturable, unidirectional system, and the saturation in this transport mechanism could lead to leptin resistance [62]. Despite these apparent discrepancies, the general consensus is that leptin’s main effect is to decrease blood glucose levels, as shown in ob/ob mice and insulin depleted animals. In fact, leptin signaling in the CNS can reverse the glucose and lipid metabolic irregularities observed in animal models lacking insulin [43]. It is likely that this effect is exerted by a combination of effects at different peripheral tissues as well as at the central level [27].

### 2.3. Leptin and Lipid Homeostasis

As stated before, the relevance of endogenous leptin in lipid homeostasis is beyond any doubt since lack of leptin is associated with dyslipidemia and reversal of this alteration is more easily accomplished following leptin replacement than with restricted feeding. Comparable to glucose regulation, the effects of leptin on lipid homeostasis are quite complex and exerted at a variety of different tissues, direct or indirectly, through neuronal pathways; including the intestinal mucosa (lipid absorption) [63], muscle (increasing growth and fatty oxidation and decreasing TG) [64], pancreas (hormone secretion) [65], kidney (decreased lipid accumulation), WAT (inhibiting insulin receptor activation), BAT (lipid oxidation) [45] or the liver. In some instances, the effects of leptin on these tissues can be mediated by acting at both central and peripheral levels. The relative contribution of each one is far from being understood since it may vary in relation to the metabolic challenges faced (see review [44]). In this manuscript, we will try to summarize the more relevant effects of leptin on lipid metabolism within the liver, exerted direct and indirectly via CNS.

Leptin prevents lipogenesis and activates fatty acid β-oxidation [39,66,67]. For hepatic inhibition of lipogenesis, leptin diminishes the expression of genes involved in the fatty acid synthesis including, sterol regulatory element-binding transcription factor 1 (SREBP1) [41,67,68] along with acetyl-coenzyme A-carboxylase (ACC), fatty acid synthase (FASN), and stearoyl-coenzyme A desaturase-1 mRNA (SCD-1) expression [41]. Likewise, after leptin treatment, ob/ob mice showed decrease elongation of very-long-chain fatty acids protein 5 (ELOVL5), an elongase protein whose over-expression has been observed to cause hepatic accumulation of monounsaturated fatty acids. Indeed, this effect seems, at least in part, independent of leptin’s ability to reduce food intake and body weight [69]. Moreover, leptin induces fatty acid β-oxidation in the liver through the induction of gene expression of proteins involved in this process, primarily mediated by the regulatory factor peroxisome proliferator-activated receptor α (PGC1α) increased expression [67]. Besides, fatty acid β-oxidation is also induced through a central leptin signaling mechanism that leads to the stimulation of hepatic AMP-activated protein kinase [70,71]. All these data suggest that a physiologic role of leptin during overnutrition is to preserve from the detrimental consequences of lipid overaccumulation. A piece of evidence in support of this antisteatotic function was the in vivo demonstration in leptin-unresponsive fa/fa ZDF rats exhibiting transgenic overexpression of the wild-type leptin receptor in their livers prevented the severe hepatic steatosis and hypertriglyceridemia that otherwise occurred [72]. In agreement with these data, the antisteatotic effects of metformin treatment in mice seem to be mediated by an increase in leptin sensitivity within the liver [73]. If during overnutrition, leptin tries to prevent the lipid overaccumulation in the liver under intense starvation conditions (48 hr of fasting), its role is key to promote a shift from carbohydrate to fat/ketone oxidation and maintain glucose homeostasis by increasing hepatic acetyl-CoA content. The reduction of hepatic glycogen observed in the starved state induces hypoleptinemia leading to a hypothalamic-pituitary-adrenal axis activation that promotes WAT (white adipose tissue) lipolysis. The endpoint of this process will be the development of hepatic steatosis, increasing hepatic DAG (diacylglycerol) and hepatic PKCε (protein kinase C-epsilon) activation. Although these results are highly interesting, they should be considered with caution since the fasting period was very prolonged [74]. More recently, Zhao et al. have shown in different animal models that in the context of obesity and leptin resistance, a partial leptin reduction after intraperitoneal (IP) treatment with anti-leptin antibodies reduced significantly liver steatosis probably due to an increase in leptin sensitivity in the hypothalamus [75].

A key mechanism to prevent liver steatosis is to increase the secretion of TG packaged as very-low-density lipoproteins (VLDL) since fatty liver appears when lipid uptake and de novo synthesis exceed the hepatic capacity to metabolize/export triglycerides [76]. Some studies have shown that peripheral leptin administration to lean animals, but not in DIO (diet-induced obesity) rats, decreases the hepatic levels and the secretion rate of TG into VLDL [66]. Moreover, it seems that leptin has an additive effect along with insulin in these suppressive effects on hepatic and systemic VLDL metabolism by stimulating depletion of liver TGs and increasing oxidative metabolism [77]. This is consistent with the finding of the decreased triglyceride-rich lipoproteins in transgenic skinny mice overexpressing leptin [78] and with the increased secretion of VLDL in mice lacking specifically leptin receptor in the liver [40]. Moreover, the loss of leptin signaling in the liver was also associated with a substantial increase in lipoprotein lipase activity, which contributes to increasing lipid droplets in the liver [40]. Interestingly, it has also been reported that after 8-days of subcutaneous leptin treatment of ob/ob mice, these presented an increased lipid export by the liver. The marked increase in VLDL secretion observed was accompanied by an inhibition of the hepatic substrate oxidation system. According to these data, the anti-steatotic effects of leptin could be due to a reduction in liver metabolic activity and the delivery of lipids to other tissues to be metabolized [69]. Besides these direct effects on the liver, leptin controls hepatic lipid metabolism through central pathways. In this context, Hackl et al. [79] have recently shown that brain leptin protects from steatosis by promoting hepatic triglyceride export and suppressing hepatic de novo lipogenesis (DNL), independently of changes in body weight and food intake. After ICV stereotaxic infusion of leptin in the brain of male rats, it was observed an effect on liver lipid metabolism; however, intraperitoneal leptin infusion had no effect on hepatic lipid content. Therefore, leptin may play a role in the incorporation of triglycerides into VLDLs; hence in systemic lipid metabolism, not only through leptin specific actions in the liver but also through its signaling in the central nervous system via hepatic vagal innervation [79].

## 3. Leptin in Non-Alcoholic Fatty Liver Disease

The liver, along with the WAT, forms a metabolic axis that plays a central role in systemic energy homeostasis by controlling the availability of energy sources needed to be used by other organs, such as skeletal muscle, brain or heart, mainly under fasting and in other catabolic states [80]. Lipids, the most energetic coin, are continuously transported either from adipose tissue to the liver and skeletal muscle or from the liver to adipose tissue and skeletal muscle [44]. This fat-liver-muscle interrelationship is essential for the regulation of glucose and lipid metabolism [81] Indeed, the powerful effect that adipose tissue lipolysis has on lipid accumulation in the liver is exemplified in studies proving that a 16 h-fasting in mice leads to a rise in serum non-esterified fatty acids that induce almost a 3-fold increase in hepatic lipid accumulation [44,82]. Due to the interplay between these two organs, a malfunction of either could lead to pathophysiological conditions affecting the other [80]. For instance, lipodystrophies can contribute to the ectopic accumulation of lipids in the liver [83]. Likewise, the unhealthy expansion of a dysfunctional WAT because of impaired capacity to adapt and expand in response to surplus energy could also contribute to the development of NAFLD [32,84,85]. In addition, increased rates of DNL are normally linked to the most common metabolic diseases [86]. Considering all the mentioned, it is easy to understand that IR, obesity and metabolic syndrome usually appear as underlying factors in NAFLD and that those pathologies are found associated very often [87].

NAFLD is a disorder defined by excessive fat accumulation in the liver (>5% of hepatocytes histologically) in the form of TG (steatosis) [87,88] and in the absence of excessive alcohol consumption or other known liver pathologies [87,89]. In a subgroup of NAFLD patients, in addition to the excessive hepatic fat, there may be an evolution to necroinflammation with varying stages of fibrosis, a condition named NASH [88,90]. Simple steatosis is considered a benign condition, reversible, asymptomatic and with few clinical complications [91]; however, it is the starting point for the progression to NASH, with the subsequent risk increases of cirrhosis, liver failure and hepatocarcinoma.

In the pursuit of understanding the disease pathogenesis, a great effort has been made for the last three decades. Several animal models have been designed and studied. Currently, they can be subdivided into three groups, dietary models, genetic models and combined dietary-genetic models. In many of these models, it can be observed that the interplay between WAT and liver is of great importance in order to avoid the development of NAFLD [92]. For instance, three of the genetic models of NAFLD have mutations in either leptin (ob/ob mice) or its receptor (db/db mice and fa/fa Zucker rats) and develop massive obesity, type 2 diabetes, dyslipidemia and the aforementioned fatty liver [92,93,94].

### 3.1. Leptin in Liver Steatosis

Hepatic steatosis is a prerequisite for NAFLD diagnosis [87]. Under physiological conditions, intrahepatic content of triglycerides results in low steady-state concentrations due to a precise balance between acquisition (uptake of free fatty acids from the plasma and DNL) and disposal of triglycerides (fatty acids oxidation and triglycerides export through very-low-density-lipoproteins) [95]. Any imbalance between lipid acquisition and removal may lead to the hallmark of NAFLD: triglycerides accumulation in the cytoplasm of hepatocytes [95,96]. Fatty liver develops in cases of defective leptin signaling as it happens in ob/ob mice, db/db mice and fa/fa Zucker rats [92,93] and in cases of lipodystrophy, in which leptin levels are extremely low [80,97]. Besides these direct effects as a result of a deficiency of leptin action within the liver, any disturbance in the hypothalamic leptin signaling can have important consequences in glucose and lipid metabolism, and finally also to contribute to liver steatosis development [20,27].

Leptin, as an anti-steatotic hormone, prevents lipids from being accumulated in the liver and promotes their mobilization. This leptin action can be easily understood by the fact that its administration to ob/ob and lipodystrophic mice prevents hepatic steatosis [39,97]. Leptin exerts one of these anti-steatotic mechanisms by regulating the transcription factor ChREBP (carbohydrate responsive element binding protein), which is a key component of lipid synthesis in the liver [98]. Hence, while ob/ob mice present high levels of ChREBP expression in the liver, a liver-specific inhibition of this factor in these animals ameliorates hepatic steatosis by a significant reduction of the lipogenesis [99]. As it was already mentioned above, leptin exerts its functions in the liver, both at a central level, via a brain-vagus-liver axis [79], and in a tissue-specific manner [44]. Thus, physiological leptin levels are necessary to prevent hepatic steatosis. However, under certain circumstances, such as diet-induced obese rats, leptin may not be capable of alleviating hepatic steatosis, probably due to leptin resistance [66,100]. The mechanisms underlying leptin resistance are still unclear, but it has been proposed, among others, that an increase in fructose intake results in an inhibition of STAT-3 (signal transducer and activator of transcription 3), abrogating leptin-mediated peroxisome proliferator-activated receptors alpha (PPARα) activation resulting in decreased fatty acid oxidation, increased SREBP-1 expression and increased hepatic triglyceride content [101].

Finally, it is worth mentioning that during the latest years, a new fascinating research field has tried to understand the possible role of circadian clock genes in the metabolism of NAFLD. Desynchrony of circadian rhythms induces lipid accumulation in the liver and promotes the progression to NASH [102,103]. In this context, disturbances in the physiological clock seem to be linked to leptin resistance and ER stress in the hypothalamus [104]. Hence, leptin signaling could be a relevant link between circadian dysfunction and metabolic abnormalities within the liver [105].

### 3.2. Leptin in Non-Alcoholic Steatohepatitis (NASH)

The mechanisms by which the progression from hepatic steatosis to steatohepatitis occur are still an important subject of research. Nowadays, it is recognized that in the evolution of NAFLD there may be involved several factors such as lipotoxicity of adipose tissue, alterations in gut microbiota, dietary factors or genetic and epigenetic factors [106,107]. For instance, liver steatosis may progress to steatohepatitis, among other mechanisms, when the hepatic accumulated lipids undergo incomplete oxidation, generating elevated concentrations of toxic metabolites that damage hepatocytes and could lead to cell apoptosis. This attracts and activates inflammatory cells to later progress to liver inflammation and, ultimately, to fibrosis if the inflammatory process becomes chronic [108,109].

Regarding this, a well-established knowledge involves leptin in the modulation of immunity and inflammation [110,111]. Leptin may protect from the accumulation of lipids in the liver; however, if the disease progresses, leptin may worsen it by acting as an inflammatory and fibrogenic factor [12,112,113]. A plausible explanation for this is that leptin-dependent induction of fatty acid oxidation and mitochondrial respiration may produce a state of oxidative stress. Interestingly, leptin also induces redox gene expression, probably to restore intracellular redox homeostasis and avoid a chronic inflammation process [67].

Leptin was shown to be required for hepatic fibrosis development in response to chronic liver injury [71,114]. Leptin-deficient mice failed to develop fibrosis during steatohepatitis or in response to chronic toxic liver injury; they even failed to upregulate collagen-I expression. Indeed, liver fibrosis was only recovered by exogenous administration of leptin, but not by the correction of the obese phenotype by dietary manipulation. Moreover, leptin deficiency does not alter hepatic tumor necrosis factor (TNF) regulation, but leptin is necessary for the induction of bioactive transforming growth factor β 1 (TGFβ1) protein in the context of chronic liver injury [114]. Moreover, a leptin-induced upregulation of this growth factor caused both hepatocellular carcinoma and cholangiocarcinoma via a dominant ERK (extracellular signal-regulated kinase) pathway in a zebrafish animal model [115]. Another example of leptin contribution to inflammation and fibrosis can be found in the NASH progression and increased expression of CD14 (an endotoxin coreceptor recognizing bacterial lipopolysaccharide) observed in high-fat diet-induced steatosis mice, but not in their respective chow-fed-control mice. Imajo et al. concluded that leptin significantly increases Kupffer cell CD14 expression via activation of STAT3 signaling, which in the liver results in a hyper-inflammation response to gut-derived low-dose bacterial endotoxin and in the progression from simple steatosis to steatohepatitis with liver inflammation and fibrosis. Indeed, when leptin was co-administrated in chow-fed mice also induced the upregulation of CD14 in Kupffer cells, which could lead to hepatic inflammation and fibrosis in the absence of steatosis. However, db/db and ob/ob mice presented a decrease in hepatic CD14 expression and a decrease in responsitivity to bacterial endotoxin despite the severe obesity and steatosis. The upregulation of CD14 by leptin-mediated signaling in Kupffer cells is critical in regulating the hepatic inflammatory response to the bacteria-mediated progression of NASH, although even in a healthy liver may exist an activation of this via irrespective of the presence of steatosis [113]. Consequently, leptin is capable of inducing inflammation and fibrosis without existing steatosis. Therefore, leptin can be considered a profibrogenic and a proinflammatory adipokine, and for that role has been extensively characterized [71,116]. Noteworthy, leptin signaling deficient mice, ob/ob and db/db showed enhanced intestinal permeability that makes liver cells more sensitive to bacterial endotoxins and can contribute to the hepatic inflammatory process [117]. The intestinal barrier dysfunction under aberrant leptin signaling has been associated with the hyperglycemic state characteristic of the metabolic syndrome and related diseases [118]. To summarize, the gut microbiota has been proposed as an emergent player in NASH/HCC development, and leptin signaling could be a key mediator in this process [115,119].

The promotion of liver inflammation and fibrosis lead by elevated leptin in animal models may be exerted by its direct action on Kupffer cells [120,121,122,123], hepatic stellate cells (HSC) [124,125,126,127] and even on sinusoidal endothelial cells [12,16,128]. All of those cells can express LEPRs, and the activation of this receptor by leptin contributes to hepatic inflammation and fibrosis through the increase of growth factors and proinflammatory and proangiogenic cytokines [12]. The pathway or pathways by which leptin induces liver inflammation and/or fibrosis are not fully understood; however, TGF-β may play a central role in the process. Leptin upregulates the expression of TGF-β, a factor known to be important in fibrogenesis, leading to the activation of HSC, consequently increasing the fibrogenic response in the liver [112,129]. Hepatic microRNA21 (miR21), which can be upregulated by leptin-mediated upregulation of nicotinamide adenine dinucleotide phosphate (NADPH) oxidase, has been proposed as a key regulator of TGF-β signaling, and an increase of this signaling can cause fibrogenesis [128,130] by inhibiting PPARα expression [131]. Moreover, liver miR21 induction also regulates the activation of hepatic stellate cells (HSC), therefore inducing collagen deposition and increasing fibrogenesis [130]. Leptin-induced miR21 also aids in NASH development by exerting a sinusoidal endothelial injury due to a decreased functionality of nitric oxide synthase-3 [128]; a sinusoidal dysfunction is an early event in NASH progression. In the context of NAFLD, also other dysregulated miRs, small non-coding RNAs with a regulatory role in transcriptional control mechanisms critically, contribute to NAFLD progression towards more severe stages [132,133,134]. miR122 was demonstrated to inhibit liver fibrosis and reduced HSC proliferation; however, leptin treatment was able to reduce its expression in HSC both in vitro and in vivo through the hedgehog signaling pathway [135] and by inducing the phosphorylation of FoxO1 via PI3K/Akt signaling pathway [136]. Likewise, miR122 inhibited leptin-induced liver fibrosis in the leptin-deficient mouse model [135]. Considering these results, a downregulation of miR122 induced by leptin could promote NAFLD/NASH development. On the other hand, leptin also upregulated miR-27a/b-3p levels in hepatic stellate cells and increased both α-SMA and α1(I) collagen expression promoting liver fibrosis [137]. In contrast, downregulation of overexpressed miR-27a and 27b allowed activated HSCs to restore their ability to accumulate cytoplasmic lipid droplets and decreased HSCs proliferation [138]. All these findings provide the potential of using miRNAs as new promising therapeutic targets for NAFLD/NASH treatment and suitable non-invasive biomarkers for patients suffering from these pathologies [132,133,139]. However, due to the great variability of microRNAs and that their regulatory mechanisms can be highly cell/tissue-specific, future studies should answer many open-ended questions.

In HSCs, leptin upregulates GATA binding protein-2 through β-catenin and sonic hedgehog pathways, decreasing PPARγ (peroxisome proliferator-activated receptor-gamma) expression, which has been postulated to be responsible for the elevated number of activated HSCs [140]. For activated HSC, leptin is a mitogen and survival factor, preventing their apoptosis, increasing the number of fibrogenic cells [141]. Conversely, there is evidence showing that activated HSCs reciprocally contribute to leptin expression and subsequent exacerbation of liver fibrosis; in contrast, quiescent HSCs have been found to be associated with higher expression of adiponectin and low levels of leptin [71]. Moreover, leptin-activated HSCs secrete proinflammatory cytokines such as monocyte chemoattractant protein-1 that aggravate the inflammatory process [142]. Many other mechanisms lead by leptin contributing to inflammation and fibrosis take place in HSC, such as upregulation of collagen type I [143], stimulation of the production of tissue inhibitor of metalloproteinase (TIMP)-1 thus inhibiting collagen degradation [144] or the repression of gene expression of matrix metalloproteinase (MMP)-1 leading to collagen accumulation [145]. Likewise, leptin modulates the expression of other metalloproteinases that play a crucial role in liver fibrosis [13,146].

Leptin could contribute indirectly to hepatic inflammation and fibrogenesis through the increased expression of adhesion molecules that may well be responsible for the activation and migration of immune cells to inflammation sites in the liver. Specifically, leptin increases the expression of intercellular adhesion molecule- 1 (ICAM-1, CD54) and very late antigen-2 (VLA-2, CD49b) on CD4 T cells, possibly through the induction of proinflammatory cytokines such as IFN-γ (interferon-gamma) [71,147,148]. Furthermore, it has been shown in vitro that leptin can have effects on activation, proliferation, maturation and production of inflammatory mediators in several immune cells that include lymphocytes, monocytes/macrophages, dendritic cells (DC), neutrophils and eosinophils. Those cells express LEPRs, and the described effects may be mediated by the binding of leptin to its receptor with the subsequent activation of STAT-3, PI3K (phosphatidylinositol 3-kinase) and p38 MAPK (p38 mitogen-activated protein kinase) signaling pathways [71]. Interestingly, the link between leptin and mTOR (mammalian Target of Rapamycin) pathway plays an important role in inflammation responses. Indeed, rapamycin was able to inhibit leptin’s effect on macrophage lipid metabolism and inflammatory mediator production [149].

Another critical mechanism by which leptin is implicated in altered lipid metabolism and contributes to the initial development of steatosis and to NASH progression is directly related to hepatic iron metabolism, which promotes lipid peroxidation and alters insulin signaling [150]. The central regulator of iron homeostasis is hepcidin, a protein with antimicrobial properties and upregulated during inflammation by proinflammatory cytokines such as TNFα and IL6. Hepcidin is also upregulated by leptin both in vitro [151] and in vivo [152]. After leptin administration to ob/ob mice, there was a significant increase in iron content in the liver. Elevated hepatic iron concentrations could be toxic by generating hydroxyl radicals and mediating oxidative stress, which could lead to tissue damage [152]. Recently, Marmur et al. have reported that despite hepcidin levels correlate to liver iron content, there is no correlation with the degree of steatohepatitis or lipid status in patients with NAFLD [153] but a major correlation with obesity has been found [154]. However, more studies should be conducted to clarify the hepcidin role in NAFLD disease.

Finally, to notice, leptin, on HSCs, can induce the vascular endothelial growth factor (VEGF), a potent inducer of neovessel formation. As the formation of new blood vessels is relevant for wound-healing response and has been suggested to play a role in the irreversibility of established cirrhosis, these observations may have a potential impact on liver fibrosis and consequently on NASH progression [71,142].

### 3.3. Leptin in Hepatocarcinoma (HCC)

There are some well-established risk factors for the development of hepatocellular carcinomas, such as chronic hepatitis B and C, parasitic infections, heavy alcohol consumption, tobacco smoking and aflatoxins, that will be briefly cover later [155]. However, in the past years, NAFLD and obesity have also been recognized to exert a substantial risk for the development of HCC according to the “two-hit” theory [156,157] or the recently proposed “multiparallel-hit theory” [96,158]. The association between NAFLD and HCC has also been observed in non-cirrhotic livers [157], so a fatty liver per se accompanied by various inflammatory mediators such as adipokines could trigger the development of HCC. Therefore, the possible role of adipokines, such as leptin, in the promotion of tumor evolution due to a chronic inflammatory state is also plausible because of the strong association between obesity, inflammation, and HCC [110].

As we have already mentioned above, adipokines can play an active role in hepatocellular carcinogenesis development. Hence, while leptin expression has been positively correlated with HCC cell proliferation evaluated by proliferation marker protein Ki67 presence, adiponectin expression, in contrast, showed a significant correlation with the increase disease-free survival and inversely with tumor size and local recurrence [159]. Therefore, adiponectin can antagonize the oncogenic action of leptin in HCC. Noteworthy, it is suggested that women are subject to a lower HCC risk due to a lower sensitivity to leptin [160], while increased levels of adiponectin in females protect against liver cancer [161]. Moreover, estradiol and its receptors antagonize the oncogenic actions of leptin in HepG2 cells by inhibiting cell proliferation and stimulating cell apoptosis [162]. Nevertheless, the research of leptin involvement in HCC has revealed both activating and/or inhibitory effects of this adipokine over the development and/or progression of HCC [71,110,155].

The tumor suppressor activities of leptin on HCC have been associated with leptin interference with the immune system. Indeed, in a model of xenotransplantation, leptin administration significantly induced the suppression of the development of HCC. In vivo, athymic nude mice were transplanted with Hep3B human hepatoma cells, and after leptin administrations, the HCC-harboring athymic nude mice resulted in significant inhibition of the tumor growth and improved survival rates in these animals. This suppression was mediated by the induction of natural killer cell proliferation and activation [163]. Likewise, another study suggests that hepatoma cells could enhance anti-HCC immunity through secreting leptin to downregulate Treg activity and subsequently promote CD8(+) T-cell response [164]. It has also been shown in rat H4IIE HCC cells and H4IIE-derived HCC tumors that leptin inhibits HCC cell growth in vitro via a p38-MAPK-dependent signaling pathway [165]. Recently, through in vitro studies, it has been demonstrated that the inhibition of the PI3K pathway after leptin-derived peptides (OB3) treatment plays a vital role by reducing the expression of leptin-induced proinflammatory genes in hepatocellular carcinoma cells [166].

Yet, most of the evidence points at leptin to be a tumor promoter, to have negative effects on HCC. Hepatocarcinoma frequently arises in an inflammatory tumor microenvironment where interleukin-6 (IL6) is a critical mediator of HCC development through its trans-signaling pathway [167]. It is well established the crosstalk between leptin and IL6, by promoting its expression and by sharing common pathways [168,169]. In fact, it was recently observed that additional ablation of hepatic LEPR ameliorated HCC burden in total IL-6Rα deficient mice subjected to diethylnitrosamine (DEN) administration to induce liver cancer [170]. In vitro, it has been demonstrated that leptin induces the proliferation of hepatoma cells via stimulation of DNA synthesis and enhancement of mitotic activity [171] and also promotes hepatome cell invasion and migration via a signaling pathway that involves JAK/STAT, ERK, and PI3K/AKT, as it has been already mentioned throughout this manuscript [172];, but it also has a critical role in the hepatocellular carcinoma development through modulation of human telomerase reverse transcriptase [13]. Moreover, leptin exhibits an antiapoptotic effect exerted by the downregulation of Bax protein in a Janus kinase 2-dependent pathway and by upregulating cyclin D1 [173]. Leptin can also increase the proliferation and the metastatic potential of cholangiocarcinoma cells [174] and has exhibited mitogenic activities on human liver cancer HepG2 cells through the induction of methionine adenosyltransferase 2A and 2β [175]. On the other hand, significant alterations in cytokine profiles, including leptin and chemokines (C-X-C motif) ligand (CXCL1, CXCL2 and CXCL16), were observed in the plasma and liver tissue lysates from normal and steatotic mice that were further shown to directly increase hepatocyte proliferation in vitro [176]. Likewise, Ma et al. showed that the dysregulation of lipid metabolism in leptin-deficient mice causes a selective loss of intrahepatic CD4(+), but not CD8(+) T lymphocytes accelerating the hepatocarcinogenesis process [177]. Interestingly, ob/ob mice, despite lacking leptin, also present increase hepatocyte proliferative activity and inhibition of hepatocyte apoptosis. Hence, it is possible that, rather than lacking leptin per se, other metabolic abnormalities in these obese mice are mediating the hepatocyte hyperplasia [178], probably related to the role of obesity as a major risk factor for liver carcinogenesis [179].

Increased angiogenesis lead by leptin has been demonstrated in rats, where leptin-mediated neovascularization coordinated with VEGF plays an important role in the development of liver fibrosis and hepatocarcinogenesis in NASH [180]. Moreover, leptin upregulates the expression of VEGF, a potent angiogenic factor, in HSCs, via oxygen-independent activation of hypoxia-inducible factor 1alpha (HF1α) [142]. Accordingly, a lack of leptin action reduces angiogenesis and the formation of pre-neoplastic foci in experimental steatohepatitis [180]. Likewise, it has been revealed that in human HCC, LEPRs alongside with leptin, are expressed at higher levels and that poorly differentiated HCC present concomitantly higher vascularization and LEPRs expression [181].

On the other hand, in 2012, Feldman et al. provided evidence of a central role for the leptin-TISC–signaling axis in promoting obesity-induced tumor growth [182]. Tumor-initiating stem cells (TISCs) are highly malignant cells that have been identified as key drivers of malignant progression with drug resistance and share similarities with embryonic stem cells [183]. In later studies, this group identified that interactions between LPS-TLR4-NANOG and Leptin-Ob-R-STAT3 pathways are crucial to enhance TICs role in malignant tumor development and metastasis [184].

**Figure 2:**
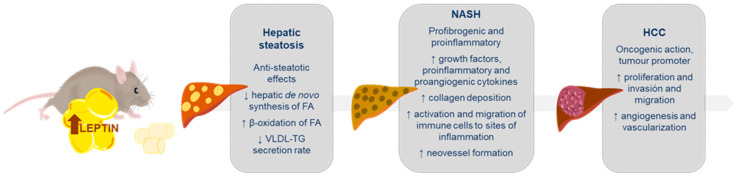
Summary showing the main changes induced by increased leptin in hepatic steatosis, non-alcoholic steatohepatitis and hepatocarcinoma. These metabolic changes can be upregulated (↑) or downregulated (↓) by the leptin action. FA (fatty acids); VLDL (very-low-density lipoproteins); TG (triglycerides); NASH (non-alcoholic steatohepatitis); HCC (hepatocarcinoma).

In conclusion, in the context of chronic liver diseases, leptin can promote proliferation, migration and invasiveness, hasten fibrogenesis, induce angiogenesis, and act directly on neoplastic cells, hence, favoring HCC development (Figure 2).

### 3.4. Leptin and NAFLD in Human Studies

Several clinical studies have tried to relate adipokines, as leptin or adiponectin, with the development and severity of NAFLD in humans. The results obtained from leptin studies are more heterogeneous and controversial than those with adiponectin. However, the characterization of serum profiles of adiponectin and leptin in human NAFLD, in general, revealed that adiponectin levels are decreased while leptin concentrations increased in NAFLD, which suggests that a misbalance of adipokines could promote the evolution of the disease [110].

Most data relating leptin with human NAFLD come from case–control studies. In patients with biopsy-proven NAFLD, Machado et al. proved that when increasing the severity of steatosis, leptin progressively increased while adiponectin decreased; moreover, leptin progressively increased with more severe fibrosis [185]. Accordingly, in a larger study, which include 1610 patients with NAFLD, it was also found that higher leptin levels were associated with increased hepatic steatosis severity based on both ultrasound findings and NAFLD fibrosis score; however, only in classic NAFLD patients the finding remained significant after adjusting for known demographic variables, this fact suggests that leptin role in NAFLD pathogenesis may be body mass index (BMI)-dependent [186]. Moreover, another study, it was also found an association between serum leptin concentration and NAFLD male and female pre-diabetic subjects; the association was mediated by insulin secretory dysfunction and IR [187]. Furthermore, the association between leptin and NAFLD in humans can also be extrapolated for the fact that two LEPR polymorphisms (Q223R and K109R) are susceptible factors for NAFLD, at least in Asian populations [188,189].

Some data can also be found in prospective cohort studies. In a 3-year prospective study with paired biopsies of NAFLD patients, it was demonstrated that in subjects with a stable or improved NAFLD activity score, the reduction in leptin levels was higher than in those with disease progression measured by an increase in NAFLD activity score; still, leptin variation could not predict the disease progression or fibrosis independently from BMI, which remained as a crucial factor associated with disease progression [190]. In another prospective study which lasted 7 years, individuals without NAFLD at baseline that developed the disease during the 7 years presented higher baseline leptin concentrations compared with those who remained NAFLD-free; however, only weight gain and higher baseline IR were found to be independent predictors for NAFLD in the 7-year follow-up [191]. Conclusively, further prospective studies, including paired-biopsies and long-term follow-up, would be more clarifying.

The complete study to date relating circulating leptin levels with NAFLD is a systematic review and meta-analysis performed by Polyzos et al. [11]. The meta-analysis included 33 studies gathering information from 2612 individuals (775 controls and 1837 NAFLD patients). Higher circulating leptin levels were observed in NAFLD patients, in patients with simple steatosis (SS) and in NASH patients than in their respective controls, and in NASH patients than SS patients. These associations remained significant after the exclusion of studies involving pediatric or adolescent populations and morbidly obese individuals subjected to bariatric surgery. This meta-analysis may render circulating leptin a factor needing re-evaluation for the use in prognostic non-invasive algorithms for NASH [11]. According to these findings, the same research group had previously shown that higher circulating leptin levels were associated with increased severity of NAFLD. They showed that NAFLD patients with a high stage of fibrosis, lobular or portal inflammation, presented higher circulating leptin levels [11,192]. Although most of the studies revealed that leptin levels have a positive correlation with NAFLD development, in a systematic review conducted in 2019, authors highlighted that these levels seem to be affected by age, gender and percentage of body fat. Consequently, so, they suggest these factors should be adjusted in future studies [193]. Under other conditions, Chitturi et al. observed that serum leptin was not an independent predictor of hepatic inflammation or fibrotic severity and that hyperleptinemia observed in NASH could not be explained simply by gender, obesity, or the presence of type 2 diabetes [194].

There are also some interventional studies in which leptin levels are measured; some of them conclude that circulating leptin levels diminish along with BMI after successful weight loss following lifestyle modifications or bariatric surgery [11,12]. Significantly, the effectiveness of the ketogenic diet to treat NALFD has been associated with an increased hepatic mitochondrial redox state and decreased hepatic citrate synthase flux due to a decreased plasma leptin concentration [195]. Similarly, vitamin E supplementation in adult patients with NAFLD resulted in a reduction of leptin levels [196]. However, studies of the effect of other drugs on leptin levels in NAFLD populations are limited and inconclusive. For instance, patients treated with rosiglitazone showed that the improvement found in liver function was not accompanied by significant changes in circulating leptin levels [197]. Similarly, a combined low dose of spironolactone plus vitamin E showed beneficial effects on patients with NAFLD, but not significant decrease of leptin levels was found in the treated group [198]. In opposition, turmeric supplementation, an Indian spice, has been proposed to be useful in the control of NAFLD complications due to its improvement in glucose indexes and the decrease in serum leptin levels that it induces [199].

Leptin has also been tested as a possible therapeutic strategy in patients with lipodystrophy, that present hypoleptinemia due to the reduced adipose tissue and NAFLD. Data from those uncontrolled studies is limited, yet, they have shown that leptin treatment decreases DNL improving hepatic steatosis and dyslipidemia [200]. Accordingly, another study showed reduced triglycerides, hepatomegaly and liver fat content after recombinant methionyl human leptin treatment [201]. Moreover, similarly, Safar Zadeh et al. demonstrated a reduction in NAFLD score due to improvements in steatosis grade and ballooning injury score after leptin treatment. However, patients with already established fibrosis experimented with no changes at this level [37]. Those observations are in agreement with the dual action of leptin observed in animals and in vitro studies; hence, leptin administration to NAFLD patients with normoleptinemia or hyperleptinemia is discouraged [202]. Nonetheless, the finding of leptin analogs that preserve the anti-steatotic profile and lack the potential inflammatory and fibrogenic actions would be of paramount importance [12] in the search for NAFLD treatments.

Moreover, serum leptin has been proposed as a tumor marker in hepatocellular carcinoma [203,204,205]. In fact, increased levels of serum leptin are a risk factor for the recurrence of stage I/II HCC after curative treatment [206]. Likewise, the positive association of LEPR expression in the cancerous lesions of HCC with the survival outcome can be explained by its inverse correlation with vascular invasion and may have prognostic value in HCC [207].

## 4. Leptin and Other Liver Pathologies

### 4.1. Liver Injury by Alcohol and Drugs

Despite the fact that NAFLD is becoming the most prevalent disease in the western world, probably because of its intrinsic correlation with other predominant pathologies such as obesity and metabolic syndrome, there are many other causes of liver disease. For instance, due to one of its main functions, detoxification, the liver also suffers heavily from the impact of alcohol and other drug consumption, and that along with virus-related hepatitis and parasitic infections, represent as well the common causes of liver disease.

As NAFLD, alcoholic liver disease (ALD) comprises a spectrum of conditions ranging from alcoholic steatosis to steatohepatitis, fibrosis and cirrhosis. Moreover, ALD progression can also increase the risk of developing HCC and causes irreparable damage [208]. As in other diseases, results obtained from animal models are crucial to decode the molecular mechanisms behind this pathology. However, most models do not recapitulate the full spectrum of human ALD, which includes: severe steatosis, hepatocellular damage and hepatic infiltration [209]. Although there are fewer studies conducted in the context of ALD, some works have tried to elucidate the leptin actions against ethanol-elicited liver toxicity. By way of illustration, it has been proved that the exogenous administration of leptin in alcohol-induced liver damaged mice results in a reduction of hepatic lipid levels, independently of changes in body weight and food intake [210]. Likewise, acute alcohol exposure in animals reduced the plasma leptin concentration in a dose-dependent manner [211]. On the other hand, leptin deficiency enhances the sensitivity of rats to alcoholic steatohepatitis. What is more, leptin resistance, present in Zucker rats (fa/fa), produced a suppression of metallothionein response after alcohol intake, resulting in enhanced lipid peroxidation and accumulation of oxidative stress in the liver [212]. Similar results were found in mouse models. After chronic alcohol exposure, mice showed leptin deficiency in association with fatty liver disease, and this phenotype was attenuated after external leptin administration. In fact, leptin administration reversed the alcohol exposure-induced liver lipid accumulation and improved dysregulated lipid metabolism hepatic genes [213]. Interestingly, recent results showed how adipose-specific lipin1 overexpression, a protein that modulates fatty acid oxidation gene expression, increased the release of leptin to alleviate alcohol-induced liver apoptosis and inflammation [214]. These results are in agreement with previous studies, where oxidative stress, cytokine secretion, cell viability and apoptosis due to ethanol exposition were reduced in human HepG2 and mouse HCC cell lines after leptin treatment [215,216]. Taken together, these data highlight the potential protective effect of leptin to cope with the deleterious effects of lipid accumulation and immune response mechanisms implicated in alcoholic liver disease.

Alcohol consumption has been shown to affect adipose tissue and leptin secretion in humans. Accordingly, patients with alcoholism present higher liver fat levels but show lower total fat mass [217] what affects leptin circulating levels. Patients with ALD have shown different serum leptin levels, but it seems clear that they present a positive correlation with both gender and body fat composition [218,219,220,221]. Indeed, some studies have reported that when BMI is considered as a cofactor, serum leptin levels were no longer correlated with the presence of cirrhosis [222]. On the other hand, some epidemiological studies suggest that lifestyle variables may affect leptin concentration. In contrast, other data show that circulating leptin levels are increased in a dose-dependent manner in chronic alcoholism, regardless of nutritional status [223]. In the context of chronic alcoholism, leptin has been proposed as a marker of alcohol-dependent-related liver damage, contrasting it with alcoholic-dependent patients’ disease without severe liver damage [224]. Noteworthy, leptin has been proved to be a key player in IR of liver cirrhosis independently of the disease etiology (alcohol consumption or virus infection), and IR is associated with a worse prognosis [225]. Regrettably, most of the studies conducted in humans are merely descriptive, so further mechanistic data and basic research will be needed to decipher the actual role of leptin in ALD.

Besides alcohol, other drugs and medical treatments can also damage the liver by an alteration in leptin secretion, causing changes in lipid homeostasis that could lead to fatty liver disease, steatohepatitis and even cirrhosis [226,227]. Promising results, obtained in animal models, support that leptin can provide protection against the lipid peroxidation characteristic of paracetamol-associated liver damage [228], probably because leptin has a direct effect on cytochrome P-450 protein family, conjugation and antioxidant enzymes that play an essential role in detoxification processes in liver microsomes [229]. The impact of leptin signaling in the participation of liver cytochrome metabolism has also been demonstrated by Larson et al. These authors have shown that cytochrome P450 1b1 (Cyp1b1), that has an important role in the oxidative metabolism of xenobiotics, likewise plays a key function in the modulation of liver fatty acid homeostasis. Its deletion in animal models was accompanied by significant suppression of liver steatosis and the lipid infiltration that precedes NASH. Interestingly, Cyp1b1 deletion increased the effects of the central leptin and growth hormone on the liver; therefore, leptin can be considered the mediator of the results observed [230]. Nevertheless, leptin also can promote fibrogenic responses in thioacetamide-induced liver injury, in part by upregulation of TGFβ [116,231,232,233]. Although in a chronic situation of liver damage, leptin could exacerbate the fibrotic state, initially, this potentially reversible process is a necessary “wound healing” response in an injured liver. This dual leptin role should be studied in depth in future projects.

### 4.2. Chronic Viral Hepatitis (CVH)

Hepatitis means inflammation of the liver. When the liver is inflamed or damaged by a viral infection, we talk about viral hepatitis. The most common types of viral hepatitis are hepatitis A, hepatitis B and hepatitis C, but we can also find hepatitis D and E, although with less incidence. Not surprisingly, animal models are very valuable and allow either complete or partial study of viral hepatitis development [234]; however, no model mimics all the characteristics present in the human disease. In this context, the concanavalin A (Con A) -induced hepatitis is a well-established model of viral hepatitis [235], and it has been demonstrated that leptin deficiency protects from Con A-induced hepatitis, independently of the nutritional state [236]. Consistently, leptin-deficient mice (ob/ob) showed less liver damage in models of T cell-mediated hepatitis [237]. In contrast, leptin resistance may increase the susceptibility to virus infection. Therefore, leptin could control the systemic immune defense by linking the nutritional status and infection response [238]. Despite the increasing evidence that claims leptin to be a part of the immunopathology of certain physiological conditions, leptin has been considered as a potential therapeutic molecule [239].

As in the context of ALD, studies conducted in humans are mainly descriptive, without an in-depth study of the possible mechanisms implicated in viral hepatitis. In CVH conditions, some studies report the levels and role of leptin in patients suffering this type of disease; however, the results are very heterogeneous. In general, the authors’ evidence that in patients with CVH, serum leptin levels increase as the degree of liver function worsens [240,241,242,243].

Some studies have reported that patients infected specifically by hepatitis C virus (HCV) that suffer from hepatitis presented high serum leptin levels [244,245,246]. Contrary to these data, it was also observed that in cirrhotic, non-cirrhotic or steatotic chronic hepatitis C (CHC) patients, serum leptin levels were not correlated with parameters of liver impairment [247,248,249,250,251]. There is also controversy regarding CHC, leptin and gender, BMI and age; while some papers showed a positive relation between serum leptin levels and female gender [240,242,250], BMI [242,244,245], and age [240,242], others manifested no relation between serum leptin levels and these parameters in steatotic patients [244], genotype 3 infected patients [242] or cirrhotic patients [245].

In conclusion, there is a great disparity in data connecting leptin and CVH be caused by different factors, the size of the cohort considered in each study, potential confounding factors or the different method of assignment. However, although the role of leptin in CVH remains uncertain, there are enough data to support that leptin is an important player in viral hepatitis.

### 4.3. Other Liver Diseases

Throughout this review, it has been highlighted the essential function of leptin in the immune response, as well as the increased susceptibility toward infectious diseases under leptin deficiency/resistance conditions, that can cause an extensive liver injury [238]. Although very little is known about the implication of leptin in parasitic liver diseases, some studies have found an interesting interplay between both and its repercussion in the liver.

**Figure 3:**
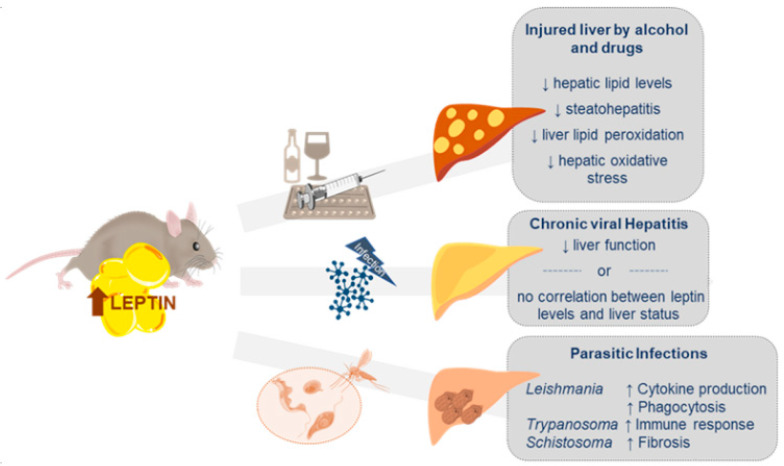
Summary showing the main alterations induced by increased leptin in the injured liver by alcohol or drugs and in the course of chronic viral hepatitis and parasitic infections. These alterations can be potentiated (↑) or decreased (↓) by the leptin action.

The liver is one of the primary target organs in visceral leishmaniasis, caused by the protozoan parasite *Leishmania donovani*. Some studies have revealed that leptin improved cytokine production and phagocytosis of this parasite by increasing the intracellular reactive oxygen species (ROS) generation both in murine and human cell lines [252]. Alternatively, ob/ob mice showed higher liver parasite burden when comparing with infected wild-type mice. Moreover, an impaired leptin signaling is linked to a reduction of the innate immune response to *Leishmania* [253], and low levels of leptin have been associated with severity parameters in visceral leishmaniasis patients [254]. Additionally, *Trypanosoma cruzi*, the etiological agent of Chagas´ disease, also causes an intense inflammatory response within the liver. Curiously, a correct regulation of metabolic parameters or immune function through central LEPR seems to be determinant against *T. cruzi* infection [255]. On the other hand, hepatic schistosomiasis represents one of the main causes of liver fibrosis worldwide. The disease is caused by embedded schistosome eggs, a genus of trematodes, that trigger an inflammation process and finally a hepatic granuloma formation [256]. Interestingly, leptin, through the TGF1β activation, has been highlighted as a potentiating factor in the development of fibrosis produced by *Schistosoma mansoni* infection. Hence, chronic leptin administration in ob/ob mice infected with the flatworm resulted in an increase in the amount of fibrosis [257]. Noteworthy, HSC activation seems to be mediating the fibrogenesis process [257,258]. However, leptin is not an essential factor since leptin deficiency reduces but does not eliminate the development of liver fibrosis in infected mice [259] (Figure 3).

## 5. Leptin Role in the Liver Regeneration Process

Liver regeneration is one of the most fascinating biological processes that has been studied by clinicians and scientists for many years. Indeed, the knowledge of the regenerative capacity of the liver dates back to the Greek myth of Prometheus and still today, the liver is the only visceral organ that shows the ability to regenerate after a partial resection and/or liver damage [260]. Despite all the studies conducted to clarify how this well-orchestrated process occurs, there are still many unknowns about how these liver repair mechanisms exactly take place. Today, we know that many signaling pathways work together in order to promote the progression of the regenerative responses while maintaining liver function [261]. Among these molecular pathways, evidence support that hepatic leptin signaling is involved in the overall hepatic regeneration process.

Considering that an injury in the liver is the first step required to trigger its regeneration, the most abundant results in this field come from studies conducted in rodent animal models either subjected to two-thirds partial hepatectomy (PHx) [262] or those of chronic injury after CCl_4_ administration at hepatotoxic concentrations [263] or dietary modifications [264,265]. In this context, it has been reported that the ob/ob mice model showing NAFLD exhibited an impaired liver regeneration after PHx or toxic damage compared to the controls [266,267,268]. This impairment manifests itself with a compromised and delayed DNA synthesis, mitosis and a nonproliferative hepatocyte phenotype. Similar results were described in db/db mice and fa/fa rats. As a case in point, when compared to their wild-type littermates, db/db mice evidenced a significant delay in the increase of the hepatic mass, reaching at 10 days a liver mass that is half that in the controls and a less pronounced mitosis index. The presence of abnormal metabolism and inhibited angiogenesis in these animal models could, at least partially, explain the results observed [269]. Another possible mechanism could implicate the decreased epidermal growth factor receptor (EGFR) in db/db fatty liver [270,271]. Likewise, fa/fa rats showed liver regeneration parameters negatively affected by steatosis after partial hepatectomy; they presented a reduction of the regenerated liver mass or a lower mitotic index. However, the steatosis per se was not enough to impair the regeneration process, given that no significant difference in proliferative response was observed after partial hepatectomy in other models of liver steatosis in rats. The deficient hepatic regeneration observed in obese fa/fa rats may be due to the lack of leptin receptor, which causes an impairment of glucose transport to hepatocytes and cells that have not enough energy to proliferate. Moreover, the availability of amino acids necessary after PHx may also be reduced [272,273]. Therefore, well-orchestrated glucose metabolism and well-balanced adipokine expression seem to be essential to prevent hepatic regeneration failures after PHx.

Interestingly, although liver steatosis is not crucial for the impairment of liver regeneration, studies reported that transient hepatic adipogenesis is a specifically regulated step of the regenerative process. Moreover, supraphysiological leptin supplementation resulted in suppression of hepatocellular fat accumulation and impairment of hepatocellular proliferation during liver regeneration in wild-type mice [274]; notwithstanding, leptin supplementation in ob/ob mice to rescue the regenerative response showed discrepancies [266,267]. This could be explained by the origin of the liver damage, the animal model phenotype or the previous liver status.

Furthermore, the administration of intraperitoneal leptin before a partial hepatectomy in Sprague Dawley rats shift to an increase of mitosis rate, regeneration ratio, and final liver weight, besides improving histopathological damage [275]. On the other hand, C57BL/6 mice subjected to a western diet for six weeks to induce simple steatosis present an improvement of hepatic regeneration after partial hepatectomy that correlates with the increase of the leptin presence in serum among other signaling factors [276]. All these data support the hypothesis that the presence or absence of leptin plays a complex and indirect role in liver regeneration. However, studies conducted in animal models organ/cell-specific leptin knockout/knockdown would provide us more information regarding the specific molecular mechanisms implicated in the regeneration process.

**Figure 4:**
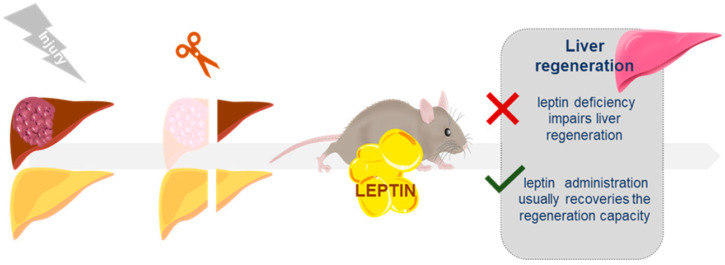
Schematic overview of leptin repercussion on liver regeneration.

In the case of acute or chronic liver failure, all the current clinical solutions are ineffective, and organ transplantation is the only treatment option. Interestingly, it was published that in healthy human liver donors, an early phase elevation of serum levels of leptin and other growth factors may be associated with accelerated liver regeneration after hepatectomy [277]. However, the limited supply of liver donors, as well as the immunological side effects, make necessary the development of alternative methods for the treatment of liver pathology. A better comprehension of the role of leptin in liver regeneration may be helpful to be applied in the regenerative medicine field (Figure 4).

## 6. Conclusions and Future Directions

There are many kinds of liver diseases and conditions, with a high prevalence and morbimortality worldwide. Currently, the major causes of liver disease are still viral hepatitis and alcohol and drug-induced liver injury, although NAFLD is increasing dramatically [278,279,280]. To be noted, parasitic infections are still neglected diseases affecting globally with an underestimated consideration, but with high levels of morbidity [281,282].

Since its discovery more than twenty-five years ago, leptin has fascinated researchers, who have tried to unravel the multiple functions where this adipokine is involved. Collectively, all the findings presented in this manuscript indicate that leptin seems to be related to NAFLD development and progression and that it plays a key role in the liver regeneration process. However, despite the large body of evidence implicating leptin in the overall scheme of hepatic diseases, the question is, friend or foe? At the same time, leptin exerts an anti-steatotic effect, decreasing lipid accumulation and lipotoxicity, and a proinflammatory effect, stimulating fibrogenesis. Probably this can be explained by a “threshold effect”, where the different leptin levels promote beneficial or detrimental consequences [283]. During the last years, scientists have put a great effort to identify the correct dosage of leptin treatment and the threshold leptin concentration in the blood, which may be effective and exert positive effects as therapeutic intervention [284]. However, given leptin´s pleiotropic activities, there is no clear conclusion yet. On the other hand, the majority of genetic animal models for studying the leptin role in liver metabolism are ob/ob and db/db mice and fa/fa rats, which have severe co-morbidities that can mask and modify some of these hepatic effects. Despite the fact that some studies have elucidated how deletions of the leptin pathway in a specific way, mainly in the CNS, can deregulate lipid and glucose metabolism in the liver, we would like to call attention to more data are needed using better conditional or inducible transgenic animal models to get specific insight into the molecular mechanism of leptin action in the context of liver diseases. Likewise, a large number of leptin targets and the different pathways in which it is implicated could explain the wide range of observed metabolic effects that ultimately allow us to adapt to the different nutritional scenarios. Regardless of the broad evidence implicating leptin in the context of NAFLD/NASH development, especially in the research with animal models, the story in humans is different [285] and more studies are needed. For instance, instead of descriptive results, interventional and controlled studies using leptin or leptin antagonists may help to clarify its role in NAFLD and to evaluate its potential use as a treatment or biomarker of the disease, either at initial steps or at more advanced levels such as NASH with or without fibrosis. Moreover, as it has been already mentioned by other authors in the past [26,286], there is still an important gap in the physiological role of leptin independently of obesity condition.

## Abbreviations

AgRP	Agoutí-related protein
AKT	Protein kinase B
ALD	Alcoholic liver disease
BAT	Brown adipose tissue
BMI	body mass index
CCK	Cholecystokinin
CD	Cluster of differentiation
ChREBP	Carbohydrate responsive element binding protein
CNS	Central nervous system
Con A	Concanavalin A
CVH	Chronic viral hepatitis
DAG	Diacylglycerol
DC	Dendritic cells
DEN	Diethylnitrosamine
DNL	De novo lipogenesis
EGFR	Epidermal growth factor receptor
ELOVL5	Elongation of very-long-chain fatty acids protein 5
ER	Endoplasmic reticulum
ERK	Extracellular signal-regulated kinase
FA	Fatty acid
FoxO1	Forkhead box protein O1
GA	Guanabenz acetate
GABA	γ-aminobutyric acid
GLP1	Glucagon-like-protein 1
GS3Kβ	Glycogen synthase kinase 3 beta
HCC	Hepatocellular carcinoma
HCV	Hepatitis C virus
HepG2	Hepatoma G2
HF1a	Hypoxia-inducible factor 1a
HPA	Hypothalamic–pituitary–adrenal
HSC	Hepatic stellate cells
ICAM-1	Intercellular adhesion molecule- 1
ICV	Intracerebroventricular
IFN-γ	Interferon gamma
IP	Intraperitoneal
IR	Insulin resistance
IRS1	Insulin receptor substrate 1
JAK	Janus kinase
LEPRs	Leptin receptors
LHA	Lateral hypothalamus area
LPL	Lipoprotein lipase
LPS	Lipopolysaccharide
MCH	Melanin-concentrating hormone
miR	MicroRNA
mTOR	Mammalian target of rapamycin
MMP	Matrix metalloproteinase
NADPH	nicotinamide adenine dinucleotide phosphate
NAFLD	Non-alcoholic fatty liver disease
NASH	Non-alcoholic steatohepatitis
NPY	Neuropeptide Y
OX	Orexin
p38 MAPK	p38 mitogen-activated protein kinase
PHx	Partial hepatectomy
PI3K	Phosphatidylinositol 3-kinase
PKCε	Protein kinase C-epsilon
POMC	Pro-opiomelanocortin
PPARα	Peroxisome proliferator-activated receptors alpha
PPARγ	Peroxisome proliferator-activated receptors gamma
SS	Simple steatosis
STAT-3	Signal transducer and activator of transcription 3
TG	Triglycerides
TGFβ1	Transforming growth factor β 1
TIMP	Tissue inhibitor of metalloproteinase
TLR4	Toll-like receptor 4
VEGF	Vascular endothelial growth factor
VLA-2	Very late antigen-2
VLDL	Very-low density lipoproteins
WAT	White adipose tissue
